# The Alberta Neonatal Abstinence Syndrome Mother-Baby Care ImprovEmeNT (NASCENT) program: protocol for a stepped wedge cluster randomized trial of a hospital-level Neonatal Abstinence Syndrome rooming-in intervention

**DOI:** 10.1186/s12913-023-09440-5

**Published:** 2023-05-06

**Authors:** Osnat Wine, Deborah McNeil, Seija K. Kromm, Karen Foss, Vera Caine, Denise Clarke, Nathaniel Day, David W. Johnson, Katherine Rittenbach, Stephen Wood, Matt Hicks

**Affiliations:** 1 Department of Pediatrics, Division of Neonatal-Perinatal Care, College of Health Sciences, Faculty of Medicine & Dentistry, University of Alberta, Edmonton Clinic Health Academy, 11405-87 Ave., Edmonton, AB T6G 1C9 Canada; 2 Maternal Newborn Child & Youth Strategic Clinical Network, Alberta Health Services, Calgary, Canada; 3 Department of Community Health Sciences, Cumming School of Medicine, University of Calgary, Calgary, AB Canada; 4 Stollery, Edmonton, Alberta Health Services, Edmonton, AB Canada; 5 University of Victoria, Victoria, BC Canada; 6 Alberta Health Services, Ponoka, AB Canada; 7 Departments of Pediatrics, Emergency Medicine and Physiology and Pharmacology, Cumming School of Medicine, University of Calgary, Calgary, AB Canada; 8 Department of Psychiatry, University of Calgary, Calgary, AB Canada; 9 Department of Obstetrics & Gynecology, Cumming School of Medicine, University of Calgary, Calgary, AB Canada

**Keywords:** Neonatal Abstinence Syndrome (NAS), Neonatal Opioid Withdrawal Syndrome (NOWS), Implementation, Culture change, Length of stay (LOS), Implementation teams, Evaluation, Opioid disorder, Prenatal substance use, Community partnerships

## Abstract

**Background:**

Neonatal Abstinence Syndrome (NAS), a problem common in newborns exposed to substances in-utero, is an emerging health concern. In traditional models of care, infants with NAS are routinely separated from their mothers and admitted to the Neonatal Intensive Care Unit (NICU) with long, expensive length of stay (LOS). Research shows a *rooming-in approach* (keeping mothers and infants together in hospital) with referral support is a safe and effective model of care in managing NAS. The model’s key components are facilitating 24-h care by mothers on post-partum or pediatric units with support for breastfeeding, transition home, and access to Opioid Dependency Programs (ODP). This study will implement the rooming-in approach at eight hospitals across one Canadian Province; support practice and culture shift; identify and test the essential elements for effective implementation; and assess the implementation’s impact/outcomes.

**Methods:**

A stepped wedge cluster randomized trial will be used to evaluate the implementation of an evidence-based rooming-in approach in the postpartum period for infants born to mothers who report opioid use during pregnancy. Baseline data will be collected and compared to post-implementation data. Six-month assessment of maternal and child health and an economic evaluation of cost savings will be conducted. Additionally, barriers and facilitators of the rooming-in model of care within the unique context of each site and across sites will be explored pre-, during, and post-implementation using theory-informed surveys, interviews, and focus groups with care teams and parents. A formative evaluation will examine the complex contextual factors and conditions that influence readiness and sustainability and inform the design of tailored interventions to facilitate capacity building for effective implementation.

**Discussion:**

The primary expected outcome is reduced NICU LOS. Secondary expected outcomes include decreased rates of pharmacological management of NAS and child apprehension, increased maternal ODP participation, and improved 6-month outcomes for mothers and infants. Moreover, the NASCENT program will generate the detailed, multi-site evidence needed to accelerate the uptake, scale, and spread of this evidence-based intervention throughout Alberta, leading to more appropriate and effective care and use of healthcare resources.

**Trial registration:**

ClinicalTrials.gov, NCT0522662. Registered February 4^th^, 2022.

**Supplementary Information:**

The online version contains supplementary material available at 10.1186/s12913-023-09440-5.

## Contributions to the literature

- An implementation study across a single-payer healthcare system of a rooming-in model of care for mothers and babies exposed to opioids during pregnancy to support the wellbeing of mother-infant dyads.
- Expected program outputs include evidence on health services’ economic benefits, care shift, and improved health outcomes of mothers and infants.
- The study will map the essential elements for the implementation process and interventions for behaviour, practice, and culture change within complex organizational settings.
- Identification of mechanisms and strategies for establishing and sustaining effective implementation, site capacity, relationships between community and acute care settings, uptake, and management of the rooming-in approach.

## Background

### Neonatal Abstinence Syndrome (NAS)

Neonatal Abstinence Syndrome (NAS), a group of symptoms that occur in newborns exposed to substances (*i.e.*, opioids) in utero, is an emerging health problem rising with the opioid crisis in North America [1]. More recently, the term Neonatal Opioid Withdrawal Syndrome (NOWS) has been coined to specifically describe opioid withdrawal in newborns. Given the challenges with polysubstance use or exposure in this study population the term NAS is used in this protocol.

The challenge of managing NAS is well described [2]. It primarily affects the central and autonomic nervous systems and gastrointestinal tract of the newborn and is commonly seen in the first 24–48 h after birth [3]. Babies with NAS may be very difficult to care for. In traditional models of care, infants diagnosed with NAS are routinely separated from their mothers and admitted to the Neonatal Intensive Care Unit (NICU) and often receive pharmacological therapy. The sequelae of NAS include costly and prolonged NICU admission, need for pharmacological management of NAS symptoms, negative impact on mother-infant bonding, decreased rates of breastfeeding, increased rates of child apprehension by child protective services, negative impact on maternal mental health, and psychological distress for families and care-providers [4, 5]. In addition, there are missed opportunities to engage mothers in Opioid Dependency Programs (ODP) or Virtual Opioid Dependency Programs (VODP) [6].

The problem is a priority because of increasing prenatal opioid use and NAS in North America and in Alberta, Canada where this study is located [7, 8]. The human, societal, and economic impacts will continue to worsen if care practices remain unchanged. Pregnant women are a unique population. Societal norms task mothers with risk avoidance as a moral requirement, including the assumption that mothers will abstain from substance use during pregnancy; however, this cannot be enforced legally and also raises complex ethical questions about power and control of women’s bodies. These social norms contribute to social stigma, legal, and health responsibilities, which may lead pregnant women who use substances to hide their status [9, 10]. Substance use in pregnancy and resultant NAS is seen in all socio-economic strata and all racial and ethnic backgrounds. However, the consequences of that use, including legal, medical, and social, may be different due to discrimination [11]. There are also significant challenges and inequities related to which mothers are screened for opioid use during pregnancy, and hence when there may be involvement by Children’s Services, and potentially child apprehension [12, 13].

Furthermore, the economic impact of NAS is substantial. In the US in 2017 there were approximately 7.3 infants with NAS per 1,000 live births [14]. Infants receiving NAS pharmacological management had mean hospital charges of $93,400 (USD) and a mean length of stay (LOS) of 23 days [15]. Additionally, out of hospital and non-healthcare related costs of NAS are substantial. In particular, rates of child apprehension are high and there is often ongoing involvement with Children’s Services [4]. While data are not available for Alberta there are published data available for Manitoba (Canada) related to the cost of a child being in care. In the year 2016/2017 the Manitoba annual child welfare budget was $514 million or $46,800 per child in care [16].

There are tangible and intangible societal and economic benefits in maintaining and promoting intact mother-infant dyads. Child development and long-term mental and physical health outcomes are critically dependent on early establishment of a nurturing relationship between mothers and their infants [5]. This in turn contributes to healthier families, less dependence on social services, and improved future economic contributions to society.

The problem of NAS in Alberta affects infants, parents, families, and health care team members, as well as the overall health system and society in general. Directly or indirectly, this problem has an impact on all Albertans. In the face of the ongoing opioid crisis, the lack of widespread adoption of evidence-based NAS care is a gap in health services that can be filled by a rooming-in model of care.

### The rooming-in model of care

*Rooming-in* is an evidence-informed model of care in managing NAS that is safe and effective [15, 17, 18] The focus is on keeping mothers and infants together throughout their hospital stay. With the rooming-in model of care, infants are cared for by their mothers in a supportive post-partum environment, avoiding the need for a long and expensive NICU admission. Mothers are provided with breastfeeding support; a standardized assessment of infant NAS symptoms by health care team members *e.g.*, *Eat, Sleep, Console (ESC)*(a tool designed to evaluate infants ability to manage withdrawal symptoms); with non-pharmacologic interventions, such as skin-to-skin care, and low stimulation environment [17–21]; ongoing social work support; an opportunity for maternal participation in an ODP; and supported transition home upon discharge in collaboration with community agencies. In addition, those involved in the provision of care to mothers and their infants with NAS receive education and support to address the stigma of drug use and its impact when caring for mothers with substance use disorder focusing on patient-centered and trauma-informed care [22, 23].

Another instrumental aspect of the rooming-in model’s long-term success is the continuum of care provided pre- and post-delivery, sometimes referred to as ‘*wraparound programs*’ [24]. Cross-sectoral collaborations that form connections between those involved with rooming-in in hospital settings and community support organizations/services is crucial to the continuum of care for mothers pre- and post-delivery [25].

Data from existing programs in Canada and the US confirm that mothers can successfully provide care in hospital with support from staff prior to transitioning home [15, 26]. Programs introduced to address NAS management challenges, like the Managing Abstinence in Newborns (MAiN) program, include family-centred care, methadone treatment, and rooming-in with mothers, found mean costs of $10,946.96 versus $44,544.17 (2014 USD) for infants who received routine NICU care. The MAiN rooming-in program was safe, effective, and reduced costs by more than 50% when using non-critical care beds compared with NICU beds as the main cost driver [27]. Multiple studies corroborate these findings, identifying that nonpharmacological strategies such as rooming-in for NAS management compared to NICU care were associated with a shorter hospital LOS and a decreased need for pharmacological treatment, thereby lowering hospitalization costs [26, 28, 29].

Supportive approaches for NAS management are practiced successfully in a few Canadian centres with the longest running example being the Families in Recovery (FIR) Program at BC Women’s Hospital in British Columbia (Canada) since 2002 [4, 30]. The program reported a significant decrease in need for pharmacological intervention for the baby (RR 0.40; 95% CI 0.20–0.78), shorter newborn LOS (RR 0.41, 95% CI 0.25- 0.65), and higher rates of being discharged home in the care of their mothers (RR 2.23; 95% CI 1.43–3.98) [4]. A study performed in Ontario, Canada estimated costs for a group of mothers and babies with NAS with no intervention to be three-fold higher than for those that received non-pharmacological intervention [28].

Specifically, the application of the ESC model of care decreased LOS and morphine doses for babies and increased breastfeeding rates [21]. A meta-analysis identified that breastfeeding is associated with reduced initiation and duration of pharmacological interventions and LOS [31]. In another small subset of studies, rooming-in also demonstrated increased breastfeeding rates and higher rates of discharge home into familial custody and was not associated with in-hospital adverse events or readmission [15].

Despite the successes seen with rooming-in programs there are still significant social barriers to providing care for families impacted by NAS. Nurses’ descriptions of their interactions when caring for women with perinatal substance use disorders and their infants have been described as problematic [32]. There is trepidation, discomfort, and judgement related to providing care to infants and families who require support for NAS. Care-providers experienced moral distress related to uncertainty and discomfort with addictions [33]. Nurses also described biases, stigma, negative attitudes, and vulnerabilities of both mothers and staff as challenges [34]. Others identified negative feelings towards patients with opioid use disorder and preferential concern for the baby over the mother’s wellbeing, recognizing a need for education and training to reduce stigma [35]. At the same time, opioid dependent women in the prenatal and postpartum period describe feeling internal stigma, shame, and guilt while also experiencing external stigma and ‘punitive like’ actions by staff [36].

Contextual barriers of providing non-pharmacologic care for NAS as identified by nurses included lack of education and resources available to staff and parents [37]. However, inter-, and intra-disciplinary coordination, flexibility in nurse staffing models, and unit architecture and layout were identified as possible facilitators [37]. Providing training and education on patient centered- and trauma-informed care may influence attitudes and decrease stigma and judgment [38, 39]. Evaluation of a comprehensive training program pilot identified an increase in knowledge and self-efficacy to provide care for substance using pregnant women [40].

### NASCENT

The Alberta Neonatal Abstinence Syndrome Mother-Baby Care ImprovEmeNT (NASCENT) stepped wedge cluster randomized study was funded to test a solution for NAS care by implementing the rooming-in model of care across the province of Alberta, Canada. In Canada in 2016/17 approximately 0.51% of all infants were diagnosed with NAS with an average LOS in the NICU of 15 days [41]. Increasing NAS rates in Alberta are consistent with Canada; preliminary data for 2011–2019 shows that the number of Alberta infants diagnosed with NAS increased from 1.52 cases per 1,000 live births in 2011/12 to 4.35 cases per 1,000 live births in 2018/2019 [42].

NASCENT is based upon the Maternal Medication Use and Neonatal Abstinence (MMUNA) model of care provided at the Grey Nuns Community Hospital in Edmonton (GNCH), Alberta since 2015 [43]. The MMUNA rooming-in program showed striking results in comparison to traditional models of NAS care including decreased NICU LOS (10.33 days to 0.82 days, *p* < 0.001); decreased need for morphine treatment (67% to 12%, *p* < 0.005); increased breastfeeding rates at discharge (22% to 77%, *p* < 0.01); and increased discharge of babies in the care of their mothers (66% to 100%, *p* = 0.01). The successful pilot has led to the continuation of prenatal and postnatal wraparound support, including rooming-in, as a sustainable expected standard of care for pregnant women with opioid-related substance use delivering at the GNCH. To date, 140 mother-infant dyads have participated in this rooming-in approach with continued success [44].

NASCENT is also informed by the Comprehensive Accessible care for Infants with Neonatal abstinence (CAIN) study. Through focus groups and individual interviews with care-providers in hospital and community settings, CAIN explored their experiences caring for infants with NAS. The overarching theme identified was ‘*Hope*’. Fostering ‘*Hope’* was key for families and care-providers working with mother-infant dyads [44].

Despite the evidence and experience gained, the practice of the rooming-in approach is limited in Alberta. It has been recognized that introducing guidelines and models of care without a detailed and adaptive implementation strategy does not lead to changes in care or improved outcomes; intervention and purposeful implementation are required [45]. Implementation of a rooming-in model requires readiness development of several components, including care teams, education and training, partnerships, and referral and communication pathways [46]. NASCENT is specifically designed to address the gap in adoption of evidence-based NAS care. The rooming-in model of care will be introduced at 8 acute care hospital sites. The intent is to support teams in developing their NAS rooming-in model of care. Informed by the Consolidated Framework for Implementation Research (CFIR) [47], the Capability-Opportunity-Motivation-Behaviour (COM-B) Framework, and the Behaviour Change Wheel [48], targeted interventions will include identifying site barriers, facilitators, and opportunities for growth, as well as capacity building and facilitation of tailored site operationalization. NASCENT will provide funds to facilitate staff education and training; support the development of provincial standardized care components; and help connect sites for mutual support and as a community of practice. A Provincial Clinical Practice Lead, funded by NASCENT, will develop and coordinate educational programming, onsite and virtual training prior to initiation, roll out of site-specific clinical protocols, virtual mentorship for site champions, care teams and individuals following implementation, and feedback to all sites.

NASCENT will support the hiring of site champions at each of the participating sites who will be instrumental to the implementation [49, 50]. Trained by the study team, they will be the point of contact between the NASCENT research team and the site implementation team. The site champions will collect data; facilitate the work of the site implementation team by introducing the rooming-in model of care through staff education, provide training, and mentorship; and coordinate feedback/evaluation sessions and surveys. Additionally, they will work closely with community organizations to identify and recruit participants as well as help facilitate transitions after discharge from the hospital. They will also liaise with other site champions to share challenges and successes.

In partnership with each unit, the NASCENT research team will identify site-specific facilitators and barriers, as well as contextual influences within and across sites. The team will establish relationships and work with site implementation teams within their unique context to identify opportunities for growth, develop tailored implementation strategies targeting barriers to implementing the intervention, and leveraging existing facilitators to support practice change.

NASCENT is the first study of its kind to implement the evidence-based rooming-in model of care for mothers and babies with NAS in a large scale, single-payer health system. The ultimate goal is that the rooming-in approach for babies with NAS will become the new normal in the province. The intervention will bring about provider-, unit-, and community-level behaviour practice, and culture change across a whole jurisdiction. Formative evaluation of the implementation process will identify essential elements that are required to support effective collaboration and implementation and will guide uptake and scale of the rooming-in intervention. Implementation of the NASCENT rooming-in model of care will decrease NICU LOS and related costs, improve the quality of care, as well as the social and health outcomes for mothers and babies.

## Methods/ design

**Fig. 1:**
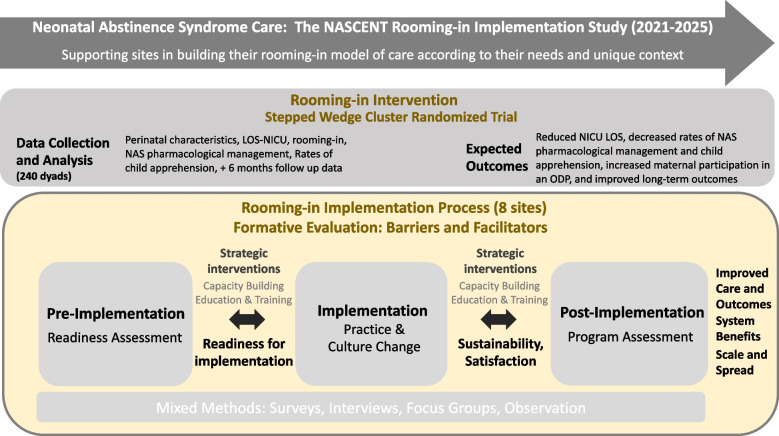
The NASCENT intervention and implementation objectives, and expected outcomes

The study has two major aims: (Fig. 1).

- **Aim 1:** Assess the impact/ outcomes of implementing the rooming-in model of care across Alberta (intervention objectives):
  - Evaluate changes in NICU LOS and the resulting economic impact (primary outcomes); and
  - Assess changes in health and social outcomes for mothers and babies (secondary outcomes)
- **Aim 2:** Implement the rooming-in model of care intervention across Alberta (implementation objectives):
  - Support the instigation/enhancement, adaptation, and sustainment of the rooming-in model of care through supporting a shift in practice and culture; and
  - Identify the elements required for implementation success: uptake, sustainability, and scale within the unique context of sites

The Consolidated Standards of Reporting Trials (CONSORT) extension for the stepped wedge randomized control [51] (Additional file 1) and the Standards for Reporting Implementation Studies (StaRI) [52] (Additional file 2) informed the preparation of the NASCENT protocol.

### The setting

Addictions and NAS have been identified as a priority health system challenge by the leadership of maternity and post-partum care units across the province of Alberta [53]. Based on approximately 56,000 live births in 2018 there are an estimated 244 affected newborns per year in Alberta. Approximately 75% of infants with NAS were admitted to the NICU with a mean NICU LOS of 14 days [53]. The mean number of infants affected and the mean NICU LOS has risen over the last 10 years in Alberta. Given this rate of increase of NAS cases it is likely that approximately 300 infants will be diagnosed with NAS yearly by 2023. In some Alberta Zones, 90–100% of infants with NAS are admitted to the NICU [53]. Maternal opioid use and subsequent NAS is not specific to large urban areas, nor is it specific to women who have been structurally marginalized, and rates of NAS per live birth are consistent across all zones and regions in Alberta [42]. NAS is common, can occur throughout all social strata, and with current care models frequently results in NICU admission, with a costly 2- to 3-week length of stay [53]. This equates to 2,500 to 3,800 NICU days per year in Alberta. Even though Alberta has a single-payer health system, the sites participating in NASCENT each operate in a unique context [*i.e.*, health services model, population served (*e.g.*, some sites serve a larger number of Indigenous women) and availability of community services for opioid dependent women pre- and post-delivery]. Additionally, a new NAS care guideline was developed for Alberta in 2019 and revised in 2022 that includes guidance on rooming-in to support the shift in care [54].

### Aim 1: Assess the impact of the rooming-in model of care intervention

#### Aim 1: design

A Stepped Wedge Cluster Randomized Controlled Trial (SW-cRCT) will be conducted with 8 participating sites in Alberta over the course of the study. The SW-cRCT is ideally suited to interventions that require implementation by multiple sites with impact on workflow and the structure of care delivery [55]. This design includes randomized sequential roll out of the intervention to all sites over time. Baseline data acts as the pre-intervention control comparisons and secular trends can be accounted for due to concurrent control groups for most periods. The analysis can also assess whether the impact of the intervention changes over time.

**Fig. 2:**
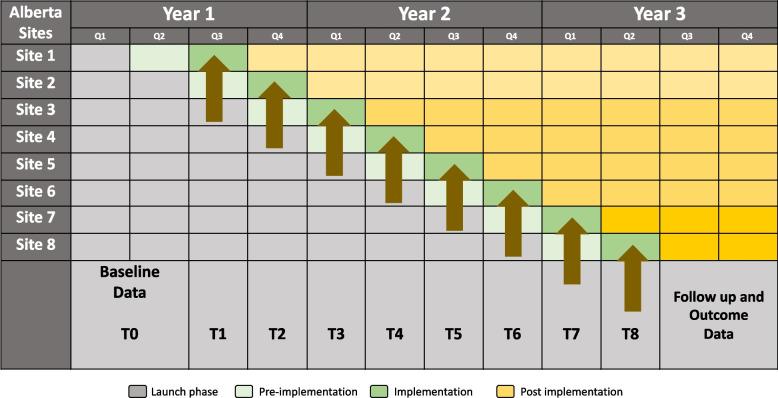
The NASCENT stepped wedge cluster design. Launch phase: baseline anonymous data collection (continues through all phases). Pre-implementation phase: formative readiness evaluation and development of tailored interventions. Implementation phase: targeted interventions, participant data collection, formative evaluation. Post implementation: formative evaluation and 6-month health and social assessment

We intend to introduce NASCENT at a new hospital every 3 months following a minimum of 6 months of initial baseline data collection as illustrated in Fig. 2. Hospitals will implement the intervention following a computer-generated stratified random allocation sequence created by the independent study statistician. Stratification based on ‘readiness’ will be used to allow the two sites with existing rooming-in programs and experience to implement early in the study to inform care at sites implementing subsequently.

#### Target hospitals

The 8 sites participating in the study all have NICU and postpartum units and some have pediatric units as well (See Additional file 3 for the full list of participating sites). Each site serves different communities and has different care structures for labour and delivery and post-partum care. Sites have a range of experience with the rooming-in model of care, from no or limited (six sites) to several years of experience (two sites). The two sites with existing rooming-in models of care programs will continue to refine and enhance their approaches and support others as they start their implementation journey.

Participating sites will be supported by the NASCENT study through the implementation process in the following ways:

- Identification and formation of Site Implementation Teams: identification of site operational, medical, nursing, and social work champions and other key individuals that need to be involved from community agencies, public health units, amongst others
- Training and support of site champions
- Identification of site-specific facilitators and barriers
- Creation of site-specific interventions and plan to prepare for NAS care, including identification of a dedicated space and commencement dates where rooming-in will take place
- Development of site-specific mechanisms to refer to an ODP/VODP program
- Development of additional staff resources to address specific learning needs
- Development of mechanisms for prenatal engagement of women who experience opioid dependency through community agencies and transition back to community
- Staff education and training (as well as funding for educational time) led by the study Provincial Clinical Practice Lead

#### Target population (mother-infant dyads)

Evaluation of the intervention outcomes will target mother-infant dyads who are admitted to rooming-in programs at one of the eight participating hospitals in Alberta. Eligibility of a mother-infant dyad to participate in the rooming-in model of care implemented by the NASCENT study/ project will be coordinated by each of the sites according to the following criteria:

- *Inclusion criteria*: Infants born at greater than 36 weeks’ gestation and a weight of greater than 2000 g born to mothers who report opioid use during pregnancy. Infants at risk for NAS must be well enough to participate in the rooming-in model of care.
- *Exclusion criteria:* Infants born at less than 36 weeks and/or birth weight less than 2000 g. Infants with congenital anomalies will be excluded because at most sites these infants would be admitted to NICU.

The goal is for site champions and study team members to recruit participants prior to hospital admission for delivery, but potential participants will also be approached if their delivery presentation is the first recruitment opportunity. During pregnancy, mothers who wish to participate in rooming-in will be identified through colleagues at community organizations. Consent to participate in the study (*i.e.,* consent to extract data using healthcare number and 6-month follow up)may be obtained prior to or immediately after delivery at the hospital.

### Data collection and management

Prospective data collection will be developed in consultation with experts from existing NAS programs and informed by the Minimum Dataset for NAS registry recommendations [56]. Anonymous LOS data will be collected from administrative data for NAS admissions at all sites pre-initiation and at each implementation phase for patients that are not consenting participants in the study. Standardized forms will be used to collect data, including LOS, for consenting participants at discharge and at 6 months follow-up (Table 1 ).

**Table 1:** Data collection: rooming-in intervention economic evaluation, and short-term social, health, and developmental outcomes

All sites	Number of Mother &Infant Dyads	Months Collected	Data
**Baseline data** (Anonymous) Throughout all phases	160–240	6 – 27	LOS in NICU, pharmacological management, breastfeeding at discharge and apprehension rates
**During implementation-** (consenting participants at/before discharge)	Up to 240	9 – 30	LOS, Perinatal characteristics, NAS pharmacologic management, rates of breastfeeding, maternal participation in ODP/VODP, child apprehension
**After discharge** (consenting participants at 6 months)	Up to 240	24	Maternal: participation in ODP/VODP, breastfeeding duration, involvement with Children’s Services, maternal depression (Edinburgh), parenting self-efficacy, quality of life Baby: NAS pharmacologic management, short-term developmental assessment using the Ages and Stages Questionnaire: SE-2 and ASQ 3

De-identified data will be collected and managed using REDCap electronic data capture tools hosted at The University of Alberta [57, 58]. REDCap (Research Electronic Data Capture) is a secure, web-based software platform designed to support data capture for research studies. In addition to chart review, provincial and national health related databases will be used to collect baseline LOS, perinatal characteristics, and data to inform the economic analysis. Data linkage will be through unique identifiers and probabilistic linkage. Data collection will be coordinated by site champions and the NASCENT study investigators. Confidentiality will be maintained throughout the study. Only study investigators will have access to the final dataset.

#### Sample size/feasibility

The mean NICU LOS over the last 10 years has consistently been 15 days (reported above) so it was selected as a conservative counterfactual and standard deviation (SD) was set at 3 days based on local and national data to provide adequate power to detect small differences in LOS. Intra-cluster correlation (ICC) was conservatively estimated at 0.1. With 8 sites, 8 initiation steps, and 1 site initiated per step (Fig. 2) over the course of 3 years, a mean LOS of 15 days (SD 3 days), and an ICC of 0.1, the study will have at least 80% power with a two-sided 5% significance level to detect a 19% difference (2.75 days) in NICU LOS.

There were 4.35 cases per 1,000 live births in 2018/2019 and approximately 56,000 livebirths in Alberta in 2018, thus an estimated 244 affected newborns are born per year. Given the rate of rise of NAS cases over the last decade it is likely that approximately 300 infants will be diagnosed with NAS yearly by 2023. Given that 75% of infants with NAS are currently admitted to NICU in Alberta, this corresponds to 225 patients being admitted to NICU by 2023.

Approximately 169 patients/year will be eligible for inclusion in the study (75% of the estimated 225 patients/year) [42]. We estimate that 4 to 6 mother-infant dyads would participate at each implementation site in each 3-month study period. Based on the 70% maternal participation rate of the MMUNA program it is estimated that approximately 20–30 mother-infant dyads would participate in a rooming-in model of care in Year 1, 90 in Year 2, and an increase to 120 dyads/year by the end of year 3, for a total of 240 expected participating dyads during the NASCENT study. 75% of participating dyads in the rooming-in model of care are expected to take part in the research (*i.e*., secondary health outcomes and 6 months follow up) considering the population involved.

### Mother-Infant dyad outcomes analysis

#### Statistical Analysis

NICU LOS was chosen as the primary outcome because NICU admission may occur for several medical reasons unrelated to NAS and a difference in LOS may be more meaningful in understanding NAS severity. Cases with no NICU admission will be assigned a NICU LOS of 0 days. Generalized Linear Mixed Models and Estimating Equations will be used in analyzing the primary and secondary outcomes: rates of pharmacological management of NAS, child apprehension, maternal participation in an ODP, maternal mental health, parenting self-efficacy, quality of life at discharge at 6 months follow up and, babies’ developmental outcomes at 6 months follow-up (see aim 1 above).

#### Economic analysis

Data extracted for the economic analysis will be used to calculate the cost avoidance related to shifting newborn care from the NICU to post-partum/pediatric units. We will compare both costs and outcomes of the NASCENT intervention with the baseline standard of care, calculating the decrease in overall NICU bed use and LOS. A significant proportion of the expected cost savings arising from this intervention do not relate to direct health system resources but include broader societal costs related to involvement with Children’s Services and decreased rates of child apprehension. These societal cost-savings will be determined using data collected from the Canadian Institute for Health Information and administrative databases. The 6 months post-discharge mother-infant dyad follow up will help ascertain short-term mother and infant health and infant developmental outcomes and will inform our economic analysis. It is also recognized that children and adolescents in care have poorer educational outcomes, higher hospitalization rates, and greater risk of attempting or committing suicide than those not in care [59–61]. These longer-term outcomes are outside the scope of NASCENT, but it is anticipated that maintaining and supporting the mother-infant dyad will have long-reaching positive impacts. A complete economic analysis and final statistical analysis for the entire study will not be performed until completion of the study.

### Aim 2: Implement the rooming-in intervention across Alberta

#### Aim 2 design

The Alberta NASCENT study is designed to assist teams in shifting from a medicalized model of care (NICU and pharmaceuticals) to a rooming-in model of care across Alberta. The overall goal is to support instigating or enhancing practice, care, and culture change through *Capacity Building* (Table 2) in each site, so it becomes the new normal practice. Capacity building is contextually driven, and measures will be taken to identify what the drivers are.

**Table 2:** Capacity Building Elements

Team and leadership	*Forming the implementation team; building a strong effective care team; establishing commitment; motivation, joint goals, responsibility, success; developing attentive leadership at units and with site champions*
Knowledge	*Founding expertise; acquiring contextual knowledge (e.g., addictions and mental health); advancing individual competencies (care providers, families/ mothers); adopting evidence-based practice and care (e.g., ESC, mother & baby-/ family- centered, trauma-informed, culturally safe care)*
Ability to change	*Changing norms of traditional care; reflecting on attitudes and beliefs; forming a unit climate supportive of learning, reflection, and evaluation*
Relationships	*Working towards trusting relationships with mothers and families; building connections within the hospital and across sectors; fostering linkages of acute care and community settings who provide care to mothers to support women pre- and post-delivery, to ensure continuum of care; ensuring open on-going channels of communication among implementation team, staff, patients, and administration*

#### Formative evaluation

Formative evaluation with care teams involved in infant care for NAS will be conducted throughout all implementation phases and will guide the development of tailored strategies to support effective implementation and bring about provider and unit-level practice change. Throughout the implementation process (launch phase, pre- during- and post-implementation), and in partnership with each unit, we will identify: site-specific facilitators and barriers, contextual influences on experiences of care teams’ interactions with women with substance use disorders and babies with NAS, satisfaction of implementation team members and participants, evaluation of interventions, and lessons learned within and across sites to articulate unique contextual needs, and potential interventions to support sustainability. Elements contributing to sustainability and retention of the rooming-in model of care including transitions to and from the hospital to community, leaders and champions, and support provided by administration will also be explored. Using theory-based surveys, interviews, and focus groups informed by CFIR [47], the COM-B Framework, and the Behaviour Change Wheel [48] we will seek to understand the implementation process and outcomes through exploration of attitudes, perspectives, and experiences to guide and attain practice and culture behaviour change (Table 3).

**Table 3:** Behaviour Change Components for NASCENT

**Practice change** Individuals, units, and hospitals	*- Establish referral processes* *- Admit NAS babies to rooming-in program instead of NICU* - *Use ESC or other methods to minimize the use of pharmacological interventions* - *Provide care for NAS babies and support mothers for longer period in hospital than usually provided for delivering mothers* - *Support mothers and families in breastfeeding and caring for the baby while in hospital* - *Ensure supports are in place in the community upon discharge*
**Culture change** Individuals	- *Health care providers provide trauma-informed care reflected as behaviours that support women to take care of their babies: e.g., listening, encouraging mothers, providing care with respect and compassion* - *Individuals’ behaviours that are NOT based on stigma and bias* - *Providing care that demonstrates cultural safety* - *Optimal care, equity based for perinatal women using substances* - *Health care providers provide hopeful care (supporting the patient’s goals and capacity to achieve their goals, and care providers’ belief in the possibility of the patient living their desired future)*

Ongoing findings will be shared with stakeholders and inform the development of tailored and strategic interventions targeting barriers and leveraging existing facilitators to improve the operation, management, and sustainability of the rooming-in model of care and enhance community involvement. Part of the planned intervention is to access existing resources to assist health care providers in examining their behaviours and identifying unconscious bias. This will help identify the elements, mechanisms, tools, and strategies to optimize the NAS rooming-in approach success (*i.e.*, uptake, sustainability, and scale of the rooming-in model of care within the unique context of sites). The NASCENT intervention plan is ideally suited to understanding and changing hospital culture and individual staff behaviour related to NAS care.

#### Participants and recruitment

To learn about each site’s unique contexts, needs, and the implementation process we will engage with the following individuals:

1. Care team involved in rooming-in for babies at risk for NAS from 8 sites, including site champions, health care providers (nurses, physicians, social workers, educators, unit mangers, etc*.*), executive directors/clinical and operational leaders, members of community agencies and public health units that work with women who use substances.
2. Mothers who participate in the rooming-in model of care at study sites will be identified by site champions or study investigators and be invited to participate.

### Data collection

There will be four phases at each site during which implementation will be assessed: Baseline, Pre-Implementation, Implementation and Post-Implementation. Data collection will aim to examine the essential elements for supporting the sites in implementing rooming-in. We will generate and collect qualitative and quantitative data at all sites from interviews, focus groups, observations, and questionnaires with health care providers and participating mothers. The questionnaires will be administered in-person, by telephone, or online through REDCap. We will provide iPads/tablets to enable participants easy access to surveys. Questionnaires will include both open- and closed-ended questions. We will seek to understand the implementation process readiness, barriers and facilitators, and experiences of care providers and parents throughout the four phases of implementation (Table 4 ).

**Table 4:** Data collection for evaluation of the barriers, facilitators, and experiences and expected activities /strategies. Evaluation methods and activities will be adapted to each site’s unique context

	How	Objective	What	Who
**Baseline**				
Evaluation	Organizational Assessment Tool (Adapted from ‘Rooming in Guideline for Perinatal Women using Substances’) [62]	-Determine timeframe for implementation Identify preliminary understanding of current practice Raise awareness and reflection on rooming-in elements	Overall competing system demands; support from hospital administration and executives; urgency; appropriate space; non-pharmacologic management of NAS; champions and leaders; staff training and education; partnerships and linkages with community and children’s services; commitment to stakeholder engagement	Hospital sites management, leaders, and key individuals involved in NAS care
	Interviews	Learn about the journey of building the rooming-in program	The initiation of the program, current practice and the lessons learned	Individuals who were involved in developing rooming-in model of care in established programs
Activities/Strategies	Introductory meetings	-Introduce NASCENT and the rooming-in approach Identify site stakeholders/ implementation team/ champion / timelines -Build relationships	1. Presentations: NASCENT overview, sharing experiences, Q&A, planning 2. Facilitation: identifying site champions, early adopters 3. Planning Sharing questionnaires findings	Hospital sites: management, leaders, and interested individuals involved in NAS care
		-Introduce NASCENT and the rooming-in approach -Build relationships	Presentations: NASCENT overview, sharing experiences, Q&A	Community stakeholders from programs and services involved in care for women with substance use
**Pre- implementation**				
Evaluation	Observation	Enable understanding of the sites’ operating practices	At working hours and site implementation meetings	Sites with an operating rooming-in model of care
	Questionnaire	Identify current knowledge, practice, attitudes, and beliefs	Individual perspectives: knowledge, expectations, barriers and facilitators, education needs, unique site context	Health care providers
	Focus group	Identify current knowledge, practice, attitudes, and beliefs	Perspectives: knowledge, unit culture, expectations, barriers and facilitators, education needs, unique context (1–2 FG depending on site size 4–6 participants)	Health care providers directly involved with caring for mothers and babies
	Interviews	Identify current knowledge, practice, attitudes, and beliefs	Individual perspectives: knowledge, experience, unit culture, expectations, barriers and facilitators education needs, unique context (4–6 interviews)	Key stakeholders, operational leadership
Activities/ strategies	Workshops/ meetings (1–2 or as needed)	Identify site implementation team, partners, current practice, context, and needs	- Current NAS practice and culture -Instigate or enhance practices - Identify and establish implementation team that will ensure continuum of care -Understand the site unique context -Asess available and required education -Identify areas of growth	Operational, medical, nursing, and social work, champions, educators, stakeholders, unit managers
		Facilitate site planning for implementation	Creation of site- plan to prepare for NAS care, *e.g.*, space, timelines, mechanisms to refer to the OPD/VOPD program, prenatal recruitment and transitions from and to community, staff resources (e.g., FAQ)	Operational, medical, nursing, and social work, champions, educators, stakeholders, unit managers
	Training	Train the trainer (site champions)	Facilitated by the Provincial Clinical Lead and the research team	Site champions
		Provide informal training	Trauma informed, anti-racist training through lived experience, peer mentoring	Health care providers with site champions educators, and peer support workers
		Advance individual and team/unit advancement: competencies, professional development, knowledge	Formal or informal learning opportunities	Health care providers with site champions educators, and peer support workers
	Education	Promote individual advancement: competencies, professional development, knowledge	Completion of staff education related to NASCENT interventions	Health care providers with site champion and/or alone (*e.g.,* modules)
		Provide education to pregnant women about the rooming-in model of care	Raise awareness and inform individuals through engagement with community organizations	Site champion Social workers Mothers
		Development of site’s tailored education plan	The Provincial clinical Lead will guide educational programming, onsite and virtual training prior to initiation, roll out of site-specific clinical protocols, virtual mentorship for care teams and individuals	Site champions and educators
	Protocols, guidelines, and FAQ	Provide standardized care	Site specific and /or across sites Provide additional support resources	Implementation teams and research team
**Implementation**				
Evaluation	Observation	Enable additional understanding of the sites’ unit culture, characteristics, challenges, and facilitators that impact implementation	At working hours and site implementation meetings	At all participating sites
	Questionnaire	Identify current knowledge, practice, attitudes and beliefs, proceeding education	Individual perspectives: training and education provided, experience, unit culture, expectations, barriers and facilitators, satisfaction	Health care providers
	Focus group	Identify current knowledge, practice, attitudes and beliefs, proceeding education	Perspectives: training and education provided experience, unit culture, expectations, barriers and facilitators, experiences (1–2 FG 4–6 participants)	Health care providers, mothers after discharge
	Interviews/ focus groups	Identify experiences	Individual perspectives: experience, unit culture, expectations, barriers, and facilities, satisfaction	Mothers after discharge
Activities/ Strategies	Planning meetings	-Identify needs, Explore facilitators and barriers -Modify interventions	-Focus on challenges and success Iterative feedback and -Continue to support capacity building through targeted interventions -Support stakeholder relationships within and outside the site	leadership Implementation team,
	Workshops	Provide education	provide ongoing staff education on identified topics	Health care providers
	Education	Increase team and individual competencies	Refinement based on evaluation	Implementation team, educator and research team
	Training	Increase team and individual competencies	-Provide learning opportunities -Refinement of training based on evaluation	Implementation team, educator and research team
	Protocols, guidelines, and resources	Provide standardized care	Refinement of existing guidelines and resources	Implementation team and research team
**Post implementation**				
Evaluation	Questionnaire	Identify current knowledge, practice, attitudes and beliefs	Individual perspectives: training and education provided, experience- lessons learned, unit culture, expectations for future practice, barriers and facilitators	Health care providers
		Identify experiences	Individual perspectives: experience, unit culture, expectations, barriers, and facilities, satisfaction	Mothers 6 months after discharge
	Focus group	Identify current knowledge, practice, attitudes, and beliefs proceeding implementation	Individual perspectives: training and education provided experience- lessons learned, unit culture, expectations, barriers and facilitators	Health care providers
	Interviews	Identify current knowledge, practice, attitudes, and beliefs proceeding implementation	Individual perspectives: experience, unit culture, expectations, barriers and facilitators	Key stakeholders, management
	Observation	Enable understanding of the sites’ unit culture and practice change	Identify challenges, and facilitators that impact implementation uptake and sustainability	At all sites
	Interviews/ focus groups	Identify experiences	Individual perspectives: experience, unit culture, expectations, barriers, and facilitators, satisfaction	Mothers after discharge
Activities/strategies	Meetings/ workshops 1 or more as needed	Planning for sustainability	Lessons learned. Barriers and facilitators to sustainment. Plans for sustainability	Care team and community partners

Legend: Implementation phases: baseline, pre- during, and post implementation

Health care providers = nurses, physicians, social workers, managers, educators, parents, and clinical and operational leaders Implementation team = those directly involved in rooming-in care. Composition will be determined by each site

Questionnaires, and focus group and interview guides will be developed by the investigative team in consultation with study collaborators, including people with lived experience. Information from interviews and focus groups will be collected by audio recording and then transcribed. Consent will be obtained for recordings before each session. Data will be in the form of transcripts, field notes, recordings, and survey responses (open and closed ended). Confidentiality will be maintained throughout the study.

### Analysis

Qualitative data from interviews, focus groups and opened questions on questionnaires will be analyzed thematically [63] by the investigative team with representation from stakeholders (member checks). Quantitative data from questionnaires will be analyzed using counts, means, standard deviations and proportions as applicable. In partnership with each site, the investigative team will identify site-specific readiness, facilitators, and barriers, as well as contextual influences within and across sites. Findings from this process that pertain to the overall site change will be mapped using the CFIR [47] constructs and domains. Expansion of the analysis that pertains to individuals’ attitudes will be mapped to the COM-B Framework [48] and then used to generate interventions, based on the Behaviour Change Wheel, to implement the rooming-in model of care. The Intervention will be adapted for each site to reflect the realities of each unit and their identified themes. The experiences and lessons learned from each site will be shared across sites to inform risk-mitigation and best-practice strategies.

### Implementation strategies

The NASCENT investigative team will establish and work with site implementation teams to jointly develop tailored implementation strategies. This joint effort will include the identification of appropriate evidence- and local experience-based adaptations to the core NAS-interventions. Existing facilitators which are known to be effective in changing practice will also be leveraged, such as recent national and provincial guidelines. We will apply targeted implementation strategies informed by COM-B behaviour change interventions and policies [48] and the Expert Recommendations for Implementing Change (ERIC) study compilation strategies [64, 65]. The strategies used will focus on building individual and team capacity through behaviour change (Table 3) to provide the rooming-in model of care effectively. We will also evaluate the effectiveness of our strategies and refine them as needed. Data collection, strategies, and activities for each implementation phase are described in Table 4. The NASCENT study is designed to establish the ‘new normal’ standard of care for infants with NAS and their mothers. Its objective is to make the education elements become part of routine training and orientation for care providers.

### Integrated knowledge translation

NASCENT is a collaboration between researchers, clinicians, the health system, and community partners. The team includes a research and leadership core group, operational leadership, and other relevant collaborators, including women with lived experience. Several committees will be established including Leadership, Implementation Science Advisory, Site Implementation Teams, Parent Advisory Board, and a committee composed of stakeholders involved in the care for women and babies outside of the hospital setting. All partners will be engaged throughout the implementation study as part of an Integrated Knowledge Translation (IKT) approach which promotes partnerships between knowledge-users and researchers in health-oriented research to help close the “know-do” gap [66].

**Fig. 3:**
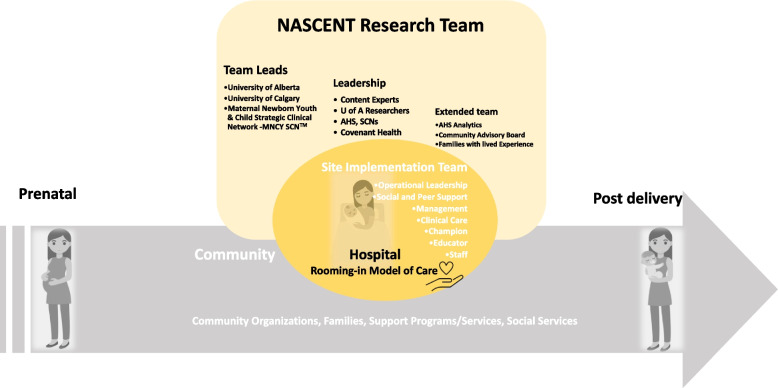
Partnerships are crucial for the continuum of care

A crucial component to the success of this implementation study is the connections/ relationships built between the hospital sites and community organizations to ensure continuum of care for mother-infant dyads (Fig. 3). Thus, support for building these relationships and continuous engagement throughout the implementation process will be instrumental.

To facilitate user and stakeholder engagement, NASCENT will support identifying and connecting with relevant stakeholders for each site; assist in forming relationships with province-wide services, programs and leaders, and practitioners; conduct preliminary meetings with relevant stakeholders to inform the upcoming program; and form spaces for collaboration and continuum of care (*i.e.*, facilitating referral pathways to hospitals that provide rooming-in care and safe transitions to the community after discharge). Moreover, stakeholders will be involved in the development of educational material to promote culturally safe, trauma-informed patient-centered care and the design of evaluation questions, analysis, and interventions.

With each step (3-month period) in the trial, data will be collected as outlined above. These data will be shared with stakeholders in feedback sessions. Specifically, information related to baseline and current rates of admission for NAS, number of participants, participation rates, LOS, and consistency of care implemented during the intervention will be fed back to sites and shared with funders. Process data related to expected implementation timeline and completion of staff training will also be collected and shared. Prospective data collection and frequent analysis (every 3 months) for each site and for the study’s overall including changes to the original plans will allow the NASCENT team to closely monitor if outcome measures are aligning with the improvement forecast. This will allow the team to regularly report actual performance metrics back to partners and funders. The study leadership team, clinicaltrials.gov, REBs, and funders will be notified of any protocol modifications. Adverse events and other unintended events potentially related to the rooming in care will be reviewed by the site implementation team and the study leadership team and reported to relevant REBs.

## Discussion

**Fig. 4:**
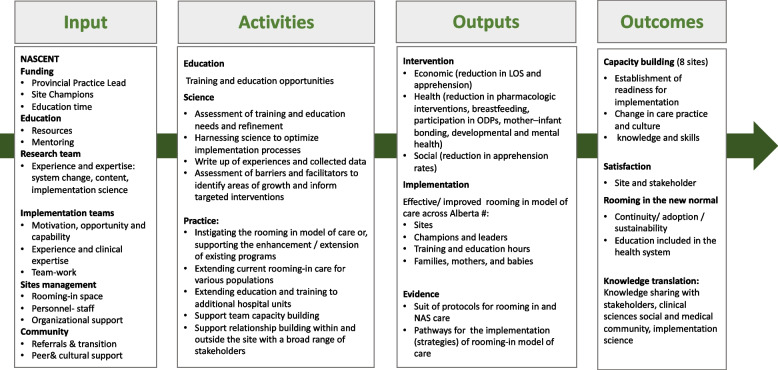
NASCENT logic model. Input, strategies, measurable outputs, and outcomes

The NASCENT study will use an evidence-based approach to implement rooming-in care for babies with NAS in all five health zones in the province of Alberta, directly addressing a priority health system challenge identified by maternal-newborn leadership across the province. The model for the NASCENT study and its associated interventions has already been tested in two sites in Alberta. NASCENT will promote the shift from traditional NAS care to a rooming-in model providing a continuum of care that supports mothers and their babies, creating a critical contact point between health services and social systems to provide mothers, who in many cases have experienced structural marginalization, with an opportunity to make life changes that will support a long-term mother and infant relationship. Moreover, NASCENT will generate the detailed, multi-site evidence needed to accelerate the scale and spread of this evidence-based intervention throughout Alberta, leading to more appropriate and effective use of healthcare resources. The ultimate goal is to demonstrate the effectiveness and efficiency of safe supportive care for mothers caring for their infants using healthcare resources more effectively and leading to more appropriate and equitable care. The NASCENT logic model portrays the input, activities and the expected outputs and outcomes that pertain to the intervention and implementation objectives (Fig. 4).

The scholarly benefits of this study will be significant. Canadian studies have identified the significant benefits of rooming-in of NAS babies with their mothers: reduced LOS, reduced pharmacological management, increased breastfeeding rates, increased acceptance and use of support services, and decreased rate of child apprehension [4, 28, 67]. The NASCENT study will evaluate all these outcomes and more on a larger scale and provide the evidence required to scale the rooming-in model of care. The NASCENT study will also empirically identify and develop implementation guidelines for NAS rooming-in including overarching facilitators and barriers on individual team and system levels, guides to identify site specific readiness indicators, needs assessment for implementation, and expected challenges and associated strategies to overcome. This study is an opportunity to collect and present a significant amount of scholarly data to the implementation, clinical science, social science, and medical communities.

### Trial status

Not yet recruiting.

## Supplementary Information

**Additional file 1. **NASCENT Protocol CONSORT Checklist.

**Additional file 2. **NASCENT Protocol StaRI checklist.

**Additional file 3.** List of participating sites across the province of Alberta, Canada.

## Data Availability

Not applicable.

## Abbreviations

NAS: Neonatal Abstinence Syndrome
NICU: Neonatal Intensive Care Unit
LOS: Length of stay
NOWS: Neonatal Opioid Withdrawal Syndrome
ODP: Opioid dependency programs
VODP: Virtual opioid dependency programs
ESC: Eat Sleep Console
MAiN: Managing Abstinence in Newborns Program
FIR: Families in Recovery
NASCENT: The Alberta Neonatal Abstinence Syndrome Mother-Baby Care ImprovEmeNT program
MMUNA: The Maternal Medication Use and Neonatal Abstinence Program
GNCH: Grey Nuns Community Hospital
CAIN: Comprehensive Accessible care for Infants with Neonatal abstinence study
CFIR: Consolidated Framework for Implementation Research
COM-B: Capability-Opportunity-Motivation-Behaviour
CONSORT: The Consolidated Standards of Reporting Trials
StaRI: Standards for Reporting Implementation Studies
SW-cRCT: Stepped Wedge Cluster Randomized Controlled Trial
REDCap: Research Electronic Data Capture
SD: Standard deviation
ICC: Intra-cluster correlation
ERIC: Expert Recommendations for Implementing Change
IKT: Integrated Knowledge Translation
