# Supplementary material for: The Alberta Neonatal Abstinence Syndrome Mother-Baby Care ImprovEmeNT (NASCENT) program: protocol for a stepped wedge cluster randomized trial of a hospital-level Neonatal Abstinence Syndrome rooming-in intervention
Source: BMC Health Serv Res. 2023 May 6;23:448. doi: 10.1186/s12913-023-09440-5 (PMC10164330; doi:10.1186/s12913-023-09440-5)
Supplement: Supplementary file 3 — Additional file 3. List of participating sites across the province of Alberta, Canada. [file 12913_2023_9440_MOESM3_ESM.docx]

**Additional file 3**

Table S1: List of participating sites across the province of Alberta, Canada (by health zone)

| **North Zone** | Grande Prairie Regional Hospital |
| --- | --- |
| **Edmonton zone** | Grey Nuns Community Hospital  Misericordia Community Hospital  Royal Alexandra Hospital |
| **Central Zone** | Red Deer Regional Hospital |
| **Calgary Zone** | Peter Lougheed Centre |
| **South Zone** | Medicine Hat Reginal Hospital |
|  | Chinook Regional Hospital |
